# Editorial: Case reports in psychopharmacology

**DOI:** 10.3389/fpsyt.2024.1364711

**Published:** 2024-01-24

**Authors:** Matej Stuhec, Patricia Di Ciano

**Affiliations:** ^1^ Faculty of Medicine, University of Maribor, Maribor, Slovenia; ^2^ Department of Clinical Pharmacy, Ormoz Psychiatric Hospital, Ormoz, Slovenia; ^3^ Institute for Mental Health Policy Research, Centre for Addiction and Mental Health, Toronto, ON, Canada; ^4^ Campbell Family Mental Health Research Institute, Centre for Addiction and Mental Health, Toronto, ON, Canada; ^5^ Department of Pharmacology and Toxicology, University of Toronto, Toronto, ON, Canada

**Keywords:** case reports, adverse events, psychiatry, clinical pharmacist, polypharmacy

## Introduction

The papers in this Research Topic reflect a range of approaches. Some have investigated the effects of monotherapy, while others explored treatments as adjuncts. Most report on psychotropics, but a few use more novel approaches, including pharmacogenetics. Many are encouraging because they describe potential new therapeutic uses of the medications. Alternatively, some case reports describe adverse events associated with the use of well-established medications. Adverse events are an important cause of medication discontinuation in psychiatry; thus, comprehensive screening is essential during treatment (1). Some involve the case of a single patient while others present the situation of a series of patients. One case report describes a collaboration with a clinical pharmacist, who provided medication reconciliation at hospital admission and discharge in a psychiatric hospital. Some countries have developed this approach, while others still lag far behind (2, 3). The range of illnesses is broad, most with a focus on psychiatric illnesses and some also on different comorbidities, including psychiatric and somatic comorbidities.

### Cases

A number of papers report on the successful treatment of schizophrenia. Jarosz and Badura-Brzoza report that the administration of combination therapy with olanzapine and zuclopenthixol was effective in reducing delusions and stabilizing mood. The treatment was well-tolerated, and sedation and extrapyramidal symptoms were not observed. Further, Renemane and Rancans explore the case of a treatment-resistant person with schizophrenia who demonstrated improvements in both positive and negative symptoms after treatment with the partial dopamine agonist cariprazine. The patient’s auto-aggressive compulsive behavior was also remitted following treatment. In a case report by Wang et al., they characterize a patient with schizophrenia who experienced significant increases in symptoms when menstruating during treatment with paliperidone extended-release tablets and olanzapine. After replacing oral paliperidone with a chemically identical, longer-lasting and more stable long-acting injection of paliperidone palmitate, she was in remission for two years.

Treatment of psychotic disorders can present a number of challenges. Logically, some papers in this Research Topic report on some of the adverse events noted during pharmacological treatment. Pjevac and Hudnik present the case of a treatment-resistant patient with schizophrenia who was treated with a number of medications: clozapine, zuclopenthixol, biperiden, flurazepam and lorazepam. The patient was believed to be experiencing anticholinergic delirium and elevated plasma clozapine level. Clozapine was gradually reduced and the dose of benzodiazepenes were lowered. The delirium gradually dissipated. Zonnenberg et al., describe the case of 5 patients with hypothermia after use of anti-psychotic treatments. They make recommendations for the assessment of the causal role of hypothermia induced by the use of antipsychotics. Preiss et al., report the case of a single patient who presented with severe hyperactive delirium after a single dose of zolpidem that was administered in combination with clozapine, aripiprazole and cariprazine. The symptom onset was rapid, occurring within a few hours. The symptoms subsided with the discontinuation of zolpidem. Torrico and Kahlon describe the case of a patient who exhibited sialorrhea after treatment with risperidone. However, concomitant treatment with clonidine reduced these symptoms, which again emerged when clonidine was removed. Levy et al., share the emergence of hyperammonemic encephalopathy after concomitant treatment with lithium and valproate semisodium for schizoaffective disorder, while Yuan et al., explore the case of a woman who developed acne after treatment with ziprasidone. Apeldoorn et al., find that buspirone worsened psychosis in a patient hospitalized for schizoaffective disorder. De Pieri et al., chart a ten year longitudinal observation of a patient with Fahr’s disease and describe the psychosis related to this disorder; this is rarely described in the literature.

The reports here also demonstrate effectiveness of pharmacotherapies in other psychiatric and neurological disorders, while also describing some of the adverse events that may occur during treatment. Guo et al., describe a case of improved depression and PTSD after initiation of augmentation with prazosin. Related to this, Richardson et al., report that the symptoms of PTSD improve even after discontinuation of 2 years of therapy with prazosin. In other work, Ha and Maguire., present a case of improvement in stuttering after initiation of treatment with deutetrabenazine, while Vayisoglu found that bupropion improved symptoms of exhibitionism. Watzal et al., characterize the emergence of pneumonitis after treatment with lamotrigine as an augmentation therapy for a mood disorder. No genetic variations in metabolizing enzymes were found to explain this relationship. Correa e Castro et al. report the case of a woman who developed compulsive buying, binge eating and hypersexuality after four years of treatment with cabergoline for prolactinomas.

Inter-individual differences in response to pharmacotherapies can play an important part in determining the response to treatment. In this regard, pharmacogenetics can be beneficial in determining the potential to respond to various treatment options. Pjevac et al., present the case of 79 year old treatment-resistant patient presenting with severe depression with psychotic symptoms. Pharmacogenetic analysis involved genotyping of CYP1A2, CYP3A4, CYP2B6, CYP2C19 and CYP2D6. Based on the patient’s genetic profile, a number of treatments were attempted. Ultimately, quetiapine and maprotiline were introduced, which results in a marked improvement in symptoms. Wu et al., describe the case of a patient who presented with serotonin syndrome despite the use of a relatively low dose of escitalopram. Gene detection revealed that she was a poor metabolizer due to her polymorphism of the CYP2C19 enzyme. Escitalopram was discontinued and the symptoms eventually resolved.

Makunts et al., describe the results of a synthesis of reports from the FDA Adverse Event Reporting System aimed at understanding some of the concomitant medications that may explain cardiovascular events associated with treatment with 3,4-methylenedioxymethamphetamine (MDMA). The authors found a number of cases of cardiovascular adverse events in which patients were taking one or more medications in addition to MDMA. There were no cases of cardiovascular adverse events associated with the use of MDMA on its own, which was is notable because MDMA increases blood pressure. In all cases, MDMA was taken with concomitant medications with known effects on cardiac function (e.g. SSRIs, antihistamine/anticholinergics, amphetamines).

Collaborative care, including a clinical pharmacist specialist, is one of the possible approaches for medication optimization. Stuhec & Batinic described two different cases where clinical pharmacists provided medication reconciliation at hospital admission and discharge in a psychiatric hospital. Clinical pharmacists recognized omitted medications and improved the transition of care. This service is reimbursed in Slovenia, and only clinical pharmacists can provide this service from 2023. In addition, clinical pharmacist optimized treatment outcomes and improved medication reconciliation. This case report should be replicated in a prospective study, including a larger effect size.In summary, these cases represent valuable information for daily practice. Further studies are needed to either confirm or reject these findings.

## Author contributions

MS: Writing – review & editing. PDC: Writing – original draft.
